# Progress of One-Dimensional SiC Nanomaterials: Design, Fabrication and Sensing Applications

**DOI:** 10.3390/nano14020187

**Published:** 2024-01-13

**Authors:** Haiyan Liu, Xiaoshan Zhang, Nana Xu, Cheng Han, Nan Wu, Bing Wang, Yingde Wang

**Affiliations:** 1 Science and Technology on Ceramic Fibers and Composites Laboratory, College of Aerospace Science and Engineering, National University of Defense Technology, Changsha 410073, China; liuhaiyan20@nudt.edu.cn (H.L.); zhangxiaoshan15@nudt.edu.cn (X.Z.); nana616849790@163.com (N.X.); hancheng@nudt.edu.cn (C.H.); wangyingde@nudt.edu.cn (Y.W.); 2 Department of Materials Science and Engineering, College of Aerospace Science and Engineering, National University of Defense Technology, Changsha 410073, China; lierenwn@nudt.edu.cn

**Keywords:** SiC, one-dimensional nanomaterials, sensors, progress

## Abstract

One-dimensional silicon carbide (SiC) nanomaterials hold great promise for a series of applications, such as nanoelectronic devices, sensors, supercapacitors, and catalyst carriers, attributed to their unique electrical, mechanical, and physicochemical properties. Recent progress in their design and fabrication has led to a deep understanding of the structural evolution and structure–property correlation. Several unique attributes, such as high electron mobility, offer SiC nanomaterials an opportunity in the design of SiC-based sensors with high sensitivity. In this review, a brief introduction to the structure and properties of SiC is first presented, and the latest progress in design and fabrication of one-dimensional SiC nanomaterials is summarized. Then, the sensing applications of one-dimensional SiC nanomaterials are reviewed. Finally, our perspectives on the important research direction and future opportunities of one-dimensional SiC nanomaterial for sensors are proposed.

## 1. Introduction

Silicon carbide (SiC), as a third-generation semiconductor material, has characteristics such as large bandgap, high breakdown voltage, and fast electron saturation drift speed. It also exhibits high-temperature resistance, oxidation resistance, acid–alkali corrosion resistance, and radiation resistance, properties that give rise to the preparation of electronic devices for use in extreme environments (such as high temperature, high radiation, and corrosive environments). It has demonstrated promise for applications such as aerospace, nuclear industry, geological exploration, and environmental monitoring [1]. At present, commercial applications of microelectromechanical system (MEMS) high-temperature sensors based on SiC semiconductor wafers have been realized. However, due to the low effective contact area between commercial SiC wafers and gases, the gas sensing response time can reach tens of seconds [2], which poses an application limitation in real-time gas monitoring and high-temperature gas leakage detection. Using nanotechnology to prepare SiC nanomaterials with high specific surface area and utilizing their abundant effective active sites are expected to solve the problem of a slow response time of commercial MEMS sensors. SiC nanomaterials are divided into zero-dimensional, one-dimensional, two-dimensional, and three-dimensional nanomaterials. One-dimensional SiC nanomaterials have unique morphology and physicochemical properties compared to zero-dimensional and two-dimensional SiC nanomaterials, which have excellent mechanical properties, anisotropic electronic transport properties, and are not prone to agglomeration, while having diverse preparation methods. It is currently the most widely studied and applied SiC nanomaterial.

**Figure 1:**
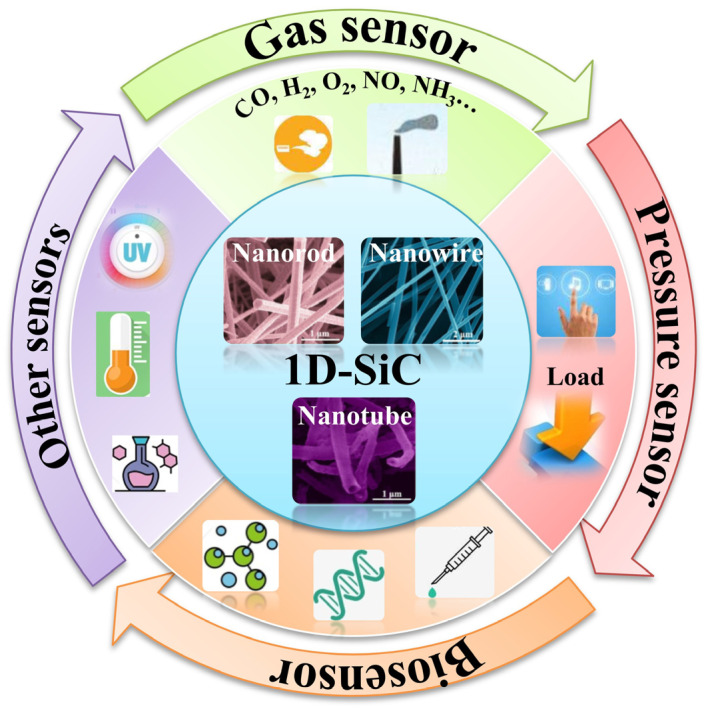
Overview of the applications of one-dimensional SiC nanomaterials in sensors.

Recently, one-dimensional SiC nanostructures (wires, rods, fibers, belts, and tubes) have become the focus of intensive research, owing to their unique application in the fabrication of electronic, optoelectronic, and sensor devices on a nanometer scale. They possess novel properties intrinsically associated with low dimensionality and size confinement, which make “bottom-up” construction of nanodevices possible [3]. In particular, SiC nanostructures are used for the reinforcement of various nanocomposite materials or as nanocontacts in harsh environments, mainly due to their superior mechanical properties and high electrical conductance. Hence, research on one-dimensional SiC nanomaterials is highlighted, both from the fundamental research standpoint and for potential application in nanodevices and nanocomposites [4]. This review focuses on the latest progress in one-dimensional SiC nanomaterials, covering the techniques for preparation and its applications in the field of sensors (Figure 1). First, a brief introduction to the structure and properties of SiC is presented. Second, the main principles and methods for designing and fabricating one-dimensional SiC nanomaterials are summarized. Third, the applications of different types of sensors are discussed. Finally, current challenges in one-dimensional SiC nanomaterial for sensors are proposed.

## 2. SiC Structure and Properties

**Figure 2:**
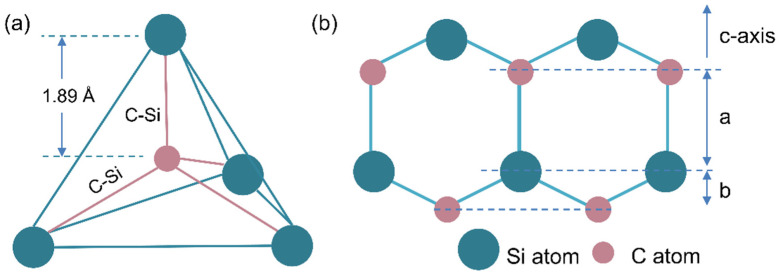
(**a**) Basic unit of SiC tetrahedron; (**b**) double-layer Si-C bond structure.

As the only stable compound of silicon and carbon, SiC has many excellent physical and chemical properties. The crystalline structure of SiC can be considered to consist of the close-packed stacking of double layers of Si and C atoms (Figure 2b). Each C or Si atom is surrounded by four Si or C atoms in strong tetrahedral sp^3^ bonds (Figure 2a). SiC has a high covalent bond energy and stable structure, but the stacking energy of C/Si double-atom layers is low, making it prone to stacking dislocation. Depending on the stacking sequence and interlayer distance, there are more than 200 polytypes in existence [5]. Polytypes can be defined by the number of stacking layers in a unit cell; the atom arrangements of popular polytypes are 3C, 4H, and 6H, and the only cubic polytype is 3C-SiC, and 4H-SiC, consisting of an equal number of cubic and hexagonal bonds. Two-thirds of 6H-SiC is composed of cubic bonds and one-third of hexagonal bonds. Only 3C-SiC is referred to as β-SiC, other 4H- and 6H-SiC are called α-SiC. In general, β-SiC, which often appears at low temperatures, is easy to nucleate and grow. However, 4H-SiC and 6H-SiC are known as high-temperature stable polytypes, which need relatively high temperatures to grow [6]. Typical properties of SiC and other semiconductors are summarized in Table 1. As compared to silicon-based semiconductor materials, SiC has a wide band gap, high carrier mobility, high electron saturation drift rate, high thermal conductivity, and high breakdown voltage. It can be used in harsh environments such as high frequency, high power, strong radiation, high-temperature corrosion, etc., which silicon-based semiconductors cannot withstand, and can meet the demand for new semiconductor materials in the military and nuclear industry.

Different polytypes of SiC have unique properties, such as breakdown electric field strength, saturated drift velocity, and impurity ionization energies. In the microelectronics industry, β-SiC is a significant material due to its high electron carrier mobility and the smallest bandgap of approximately 2.4 eV when compared to α-SiC [6]. These excellent properties make SiC a perfect material for the electronics industry, with wide applications in high-temperature, high-frequency, and optoelectronics, including rectifiers, power switches, and microwave power devices.

## 3. Preparation of One-Dimensional SiC Nanomaterials

At present, the methods for preparing one-dimensional SiC nanomaterials mainly include template, chemical vapor deposition (CVD), electrospinning, and carbothermal reduction methods. The SiC nanomaterials prepared by different methods and their applications are shown in Table 2. The various methods are described in detail below:

### 3.1. Template Method

**Figure 3:**
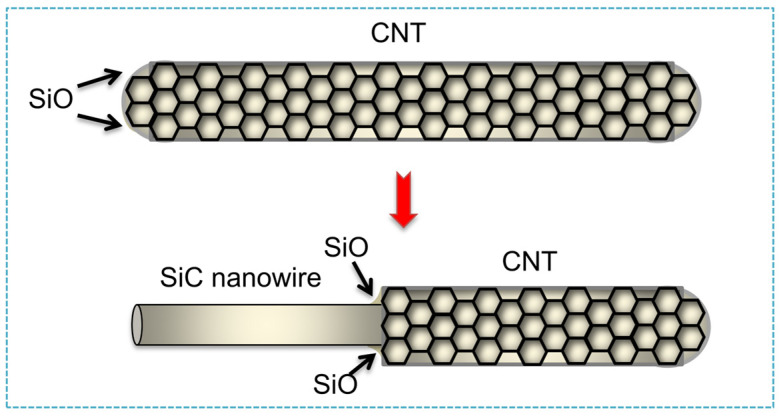
Schematic of fabrication of SiC nanowhiskers by template method.

According to the reaction mechanism, the one-dimensional SiC nanomaterials prepared by the template method can be divided into two types: one is to prepare SiC nanomaterials by in situ chemical reaction of carbon or silicon nanomaterials with silicon source or carbon source, respectively. In 1994, Zhou et al. [7] first reported the use of carbon nanotubes (CNTs) as templates to react with SiO gas for preparing SiC nanowhiskers. The results show that the unique nanostructure and high surface activity of CNTs are decisive for the growth of SiC nanowhiskers. The schematic of SiC nanowhisker fabrication by the template method is shown in Figure 3. Lieber et al. [8] and Fan et al. [22] also used CNTs with different silicon sources to prepare SiC nanorods, and the mechanical properties and optical luminescence of SiC nanorods were studied. In 2001, Ehret et al. [23,24] and Lee et al. [25] first synthesized SiC nanotubes (SiCNTs) with multiple lattice structures by precisely controlling the reaction conditions and using the shape memory effect of CNTs. Subsequently, theoretical calculations and experimental results showed that SiCNTs had a broad application prospect in hydrogen storage, gas sensing, and catalysis, etc. [26,27,28]. Recently, Ye et al. [13] fabricated a SiC@C core-shell structure by using carbon nanofibers (CNF) as a template, and then reacted with SiO at high temperatures. Afterwards, high-purity SiC nanowires were obtained by etching the CNF template.

**Figure 4:**
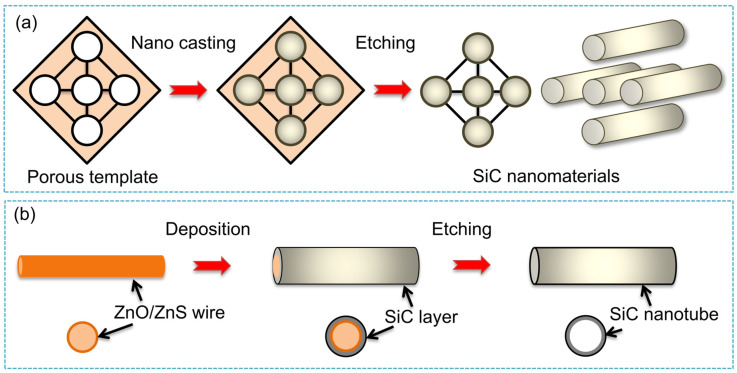
Schematics of fabrication of SiC (**a**) nanoarray and (**b**) nanotube by template method.

Another template method is first to prepare SiC on an existing ordered porous nanomaterial substrate, forming the SiC/template composite structure [29]. Subsequently, the template is etched through an acid or other solution (Figure 4). For example, by using ordered porous alumina as a template, SiC nanoarrays can be synthesized by reacting propylene [10], SiO vapor in nanopores and then etching the template (Figure 4a). A similar method is used to prepare SiCNTs, where different forms of ZnO and ZnS are used as templates to deposit SiC nanolayers on the surface through CVD [30,31]. The intermediate templates are then removed by acid etching to obtain hollow SiCNTs, as shown in Figure 4b.

The advantage of the template method is that the SiC nanomaterial with uniform morphology and diameter can be controlled by template design. However, this method is limited by the chemical reaction, and it is difficult to obtain single-crystal SiC. In addition, the process of etching and removing the template not only increases the complexity of the process but also may damage the structure of the SiC nanomaterials.

### 3.2. CVD Method

**Figure 5:**
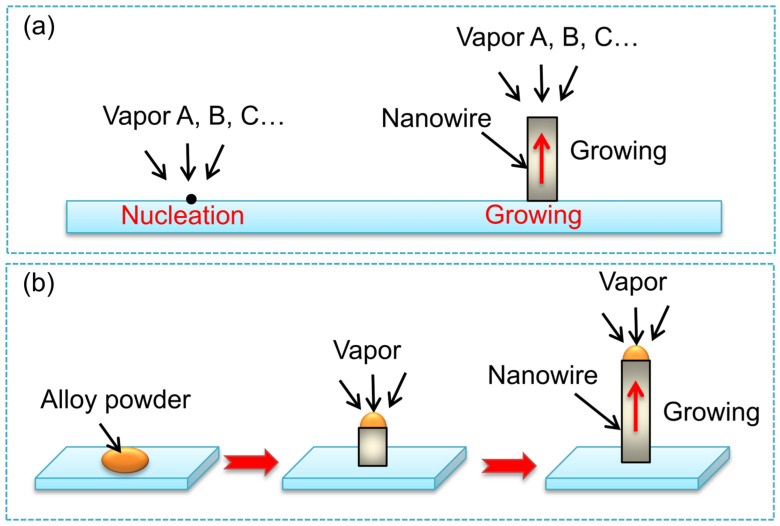
Schematic of SiC nanowires prepared by (**a**) V-S; (**b**) V-L-S mechanisms.

The principle of CVD growth of SiC nanomaterials is to vaporize silicon and carbon sources under specific pressure and temperature, and transport them to the substrate surface at a suitable speed through a certain flow of carrier gas to nucleate and grow SiC nanomaterials. As early as 1999, Zhou et al. [32] synthesized β-SiC nanowires on silicon substrates using hot-wire CVD (HFCVD) with silicon powder and graphite powder as raw materials. The nanowires had a SiC/SiO_2_ core-shell structure with a diameter of 10–30 nm and a length of less than 1 μm. Subsequently, Yang et al. [33] used CH_3_SiCl_3_ and H_2_ as reactants to prepare 3C-SiC nanowires grown along the {111} crystal plane by gas–solid (V-S) growth mechanism using CVD. The schematic diagram of the mechanism for growing SiC nanowires is shown in Figure 5a. The V-S mechanism is a classic growth mechanism commonly used to explain the growth of uncatalyzed whiskers and is now commonly used in the preparation of one-dimensional nanomaterials. At present, centimeter-scale ultra-long SiC nanowires [34], serrated nanowires [35], twin SiC nanowires [36], β-SiC/SiO_2_ nanowires [37], etc., have been synthesized and processed through the V-S mechanism. Although SiC nanowires prepared without catalysts have high purity, the morphology, scale, and crystallization direction of SiC nanowires are difficult to control, and the reaction rate is relatively slow with a low yield [38]. 

Unlike the V-S growth mechanism of SiC nanowires prepared without catalysts, the reaction rate is increased with the assistance of catalysts, and the nucleation and growth of nanowires follow the gas–liquid–solid (V-L-S) mechanism. Currently, various types of nanowires have been synthesized using catalyst-assisted CVD methods based on the V-L-S mechanism. In the general V-L-S process, the reaction begins with the dissolution of gaseous reactants in the catalyst metal nanodroplets (Fe and Ni, etc.), followed by the nucleation and growth of one-dimensional single-crystal nanostructures. The catalyst droplets play a crucial role as templates for the growth of nanowires, which can effectively control the synthesis of high-quality SiC nanowires with uniform diameter and crystallinity [39,40,41,42]. Li et al. [12] mixed liquid polycarbosilane (l-PCS), ferrocene, and carbon powder, and then pyrolyzed the mixture in an inert atmosphere at 1300 °C to prepare centimeter-length SiC nanowires by the V-L-S growth mechanism (Figure 5b). Thereby, CVD became the main method for preparing SiC nanowires, and various one-dimensional SiC nanomaterials have been synthesized using this method. Some SiC nanowires have excellent luminescent, sensing, and wave-absorbing properties, providing important references for the preparation of new structures and morphologies of SiC nanowires and the development of new functional SiC nanodevices. 

In general, the purity of SiC nanowires prepared without catalysts is relatively good, but the preparation temperature is generally high and the yield is relatively low. The addition of catalysts can significantly reduce the preparation temperature of SiC nanowires, and increase the reaction rate and yield, but it is easy to introduce impurities into the SiC nanowires. In-depth research should be conducted on improving the purity and removing impurities of SiC nanowires, while also focusing on low-cost and large-scale preparation of nanowires. Relevant measures should be taken to regulate the microstructure of SiC nanowires, and to broaden the application fields of SiC nanomaterials.

### 3.3. Electrospinning Method

**Figure 6:**
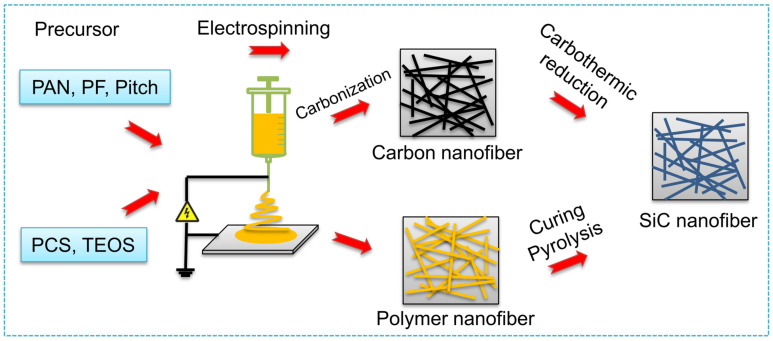
Schematic of SiC nanofibers prepared by electrospinning method.

**Figure 7:**
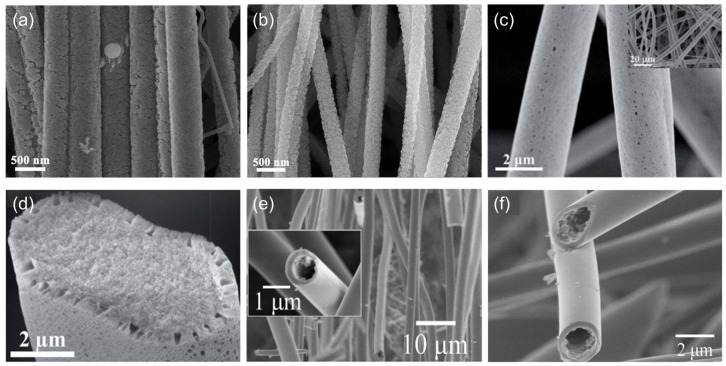
SEM images of (**a**) mesoporous SiC nanofibers [39]; (**b**) aligned SiC nanofibers [19]; (**c**,**d**) surface and cross-section of hierarchically porous SiC nanofibers [54]; (**e**,**f**) surface and cross-section of hollow SiC nanofibers [56].

At present, the preparation of one-dimensional SiC nanomaterials by electrospinning is mainly by preparing SiC nanofibers (SiCNF). A schematic diagram of the process for preparing SiCNF by different routes is shown in Figure 6. There are generally two methods, one is to use the CNF prepared by electrospinning as a template to carry out a carbothermal reduction reaction with a silicon source at high temperatures. For example, Qiao et al. [43] first coated a uniformly polymethylsilane layer on the electrospun prepared CNF, and then by curing at low temperature and pyrolysing at high temperature they obtained high crystallinity SiCNF. Cheng et al. [44] used the electrospinning method to prepare hollow CNF, and then reacted with silicon powder at high temperature to obtain hollow SiCNF. Wang et al. [14,45] prepared the mesoporous and ordered SiCNF (Figure 7a,b) by using CNF reacted with silicon powder at high temperature, and the photocatalytic hydrogen production performance of mesoporous SiCNF was carefully studied. 

The other way is to combine the electrospinning technology with the precursor conversion method to obtain SiCNF by one-step pyrolysis. Since 1976, when Yajima et al. [46] successfully prepared SiC fibers with polycarbosilane (PCS) as the precursor, the precursor conversion method has become an important method for preparing SiC fibers. In recent years, the preparation of SiCNF by the precursor conversion method combined with electrospinning has been extensively studied [47]. Eick et al. [48] first blended polycarbomethylsilane and polystyrene (PS) in toluene and dimethylformamide solvent, after electrospinning, UV curing, and pyrolysis, SiCNF with a minimum diameter of 20 nm was obtained. However, the fiber morphology and composition are unstable. Liu et al. [49] prepared SiCNF with a diameter of 1–2 nm by coaxial electrospinning using PS and PCS as raw materials, but the fiber was brittle. To improve the mechanical properties of SiC fibers prepared by electrospinning, the current measures are mainly to adjust the composition of the solution and optimize the preparation process. Yue et al. [50] and Shin et al. [51] successfully prepared micro/nano SiC fibers by increasing the ratio of low molecular weight PCS in solution. The tensile strength of the fibers after heat treatment at 1100 °C was about 1.2 GPa. Sarkar et al. [52] and Yu et al. [53] used polyaluminumcarbosilane as a precursor solution to prepare flexible hydrophobic aluminum-containing SiC fiber by the electrospinning method. Since then, studies have been ongoing to prepare SiCNFs by the precursor conversion method combined with the electrospinning method, and their application fields are being constantly expanded. Cheng et al. [15,54,55,56,57] dissolved PCS and PVP in chloroform solvent, solved the discontinuity problem of spinning by using the long-chain structure of PVP, and prepared SiC-based composite nanofibers with a diameter of 300–500 nm. The electromagnetic absorbing properties of composite fibers were studied.

Our research group has also carried out more work on the preparation of SiCNFs by the precursor conversion method. Yang et al. [58] prepared micro-nano SiC fibers using the PCS as precursor with a diameter of 0.5–2 μm by electrospinning and high-temperature sintering, and the effects of electrospinning parameters on fiber morphology were studied. Wang et al. [59] used PCS as the precursor and performed electrospinning under high humidity conditions to prepare flexible SiCNF with a hierarchical pore structure, and the gas adsorption performance and corrosion resistance of the fiber were studied. The SEM morphology is shown in Figure 7c,d. Wang et al. [60] mixed PCS with tetrabutyl zirconate and prepared a gradient structure of ZrO_2_/SiC fiber by controlling the infusibility process. The prepared ZrO_2_/SiC fiber has good high temperature and corrosion resistance. Tian et al. [61] prepared hollow SiCNF by single-needle microemulsion electrospinning. The SEM morphology of the fiber surface and cross-section is shown in Figure 7e,f. Compared with solid SiCNF, hollow SiCNF has a lower solid thermal conductivity and a higher infrared extinction coefficient, thus having a good application prospect in the field of high-temperature insulation.

In addition to PCS, polyurea silane [62,63], polydimethylsiloxane [64], ethyl orthosilicate [65], methyl triethoxy silane [66] and polymethylsilane [67] are also used as precursors to prepare SiC fibers, but their Si-C skeleton structure and ceramic yield are weaker than PCS, giving poor morphology and properties of SiC fibers. Therefore, PCS is still the most promising precursor.

Although the preparation of SiCNF by electrospinning has achieved huge development, there are still two problems to be solved: one is that the mechanical properties of fibers are generally low, or the mechanical properties and nanoscale are not compatible; Second, the prepared SiCNF has a relatively simple composition structure and cannot meet the requirements of various fields. Therefore, the focus of future research is to further improve the mechanical properties of SiCNF and develop a variety of SiCNF structures to promote its application in more fields.

### 3.4. Carbothermal Reduction Method

**Figure 8:**
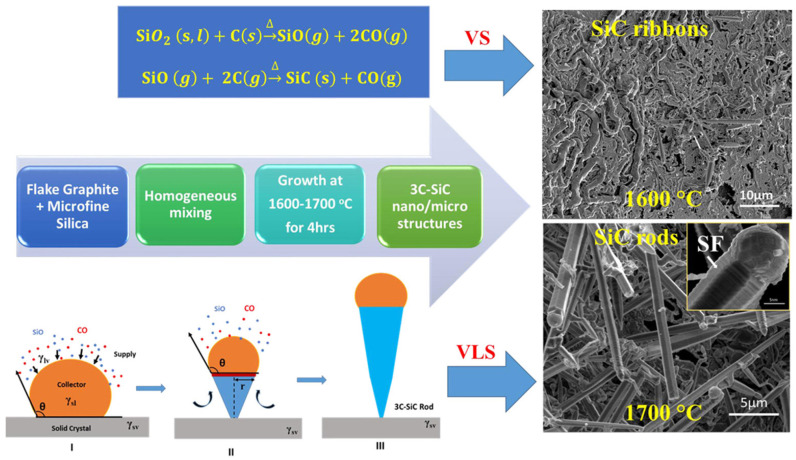
Schematic diagram of SiC nanorods prepared by carbothermal reduction method [68].

The carbothermal reduction method is to uniformly mix the silicon source and the carbon source in a solvent, form a gel after drying, and obtain a one-dimensional SiC nanostructure by a high-temperature carbothermal reduction reaction (Figure 8) [68,69]. Meng et al. [70] dissolved TEOS, sucrose, and nitric acid in an ethanol solution, and treated them at 700 °C to obtain carbon-containing silica gel, which was then reduced at 1650 °C and 3C-SiC nanowires with a diameter of 10–25 nm were obtained. Yang et al. [71] prepared mesoporous silica and sucrose as raw materials and controlled SiC nanowhisker and SiCNT by controlling the carbothermal reduction reaction temperature and holding time. The prepared SiCNT has a specific surface area of up to 190 m^2^/g. Chen et al. [72,73] formulated TEOS, hydrochloric acid, and carbon black into a sol in an ethanol solution, dried it to obtain C/SiO_2_ gel, and heated it to 1500 °C with 0.02 MPa argon gas protection to obtain cotton-like SiC nanowires. In addition, one-dimensional SiC nanostructures with different morphologies such as nanowires, multi-stage nanodisks, and nanorods can be prepared by controlling the reaction temperature and C/Si molar ratio. Maroufi et al. [17] used electronic waste as raw material, with electronic display screens as silicon source, computer plastic shells as carbon source, and prepared mesoporous SiC nanowires by pulverization, briquetting, and high-temperature pyrolysis. The obtained SiC nanowires had a diameter of 2–15 nm and a specific surface area of 51.4 m^2^/g. This method not only reduces the cost of preparing SiC nanomaterials but also provides a new idea for the secondary treatment of electronic waste worldwide. 

In addition to the above methods, there are many other methods for preparing one-dimensional SiC nanomaterials. Pei [74] and Xi [19] prepared SiC nanorods and nanobelts at 470 °C and 600 °C by the hydrothermal method and ethanol solvothermal method, respectively. Seeger et al. [75] used arc discharge technique to prepare SiC nanowhiskers. Xie et al. [20] improved this technology and used SiC rods as anode materials to achieve a large-scale preparation of SiC nanorods. Sundaresan et al. [76] prepared 3C-SiC nanowires by high-energy microwave heating and catalyst-assisted pyrolysis.

In short, the one-dimensional SiC nanomaterials have promoted rapid development of their preparation technology due to their excellent physical and chemical properties and broad application prospects. At the same time, one-dimensional SiC nanomaterials with different morphological structures have been fabricated, and the related principles fully studied. Among the many preparation methods, electrospinning technology and carbothermal reduction methods are the most promising methods for preparing one-dimensional SiC nanomaterials from the perspective of commercial scale preparation and nanostructure designability. In addition, the combination of different preparation techniques to prepare a new structure of SiC nanomaterials to meet the needs of different functional applications is an important direction for the development of one-dimensional SiC nanomaterials.

## 4. Applications of One-Dimensional SiC Nanomaterials in Sensors

As one of the most important compound semiconductors, SiC has been widely used for various sensors in harsh environments due to its wide bandgap, excellent thermal stability, high strength, good thermal shock resistance, high electron mobility, and good chemical inertness [77]. The high electron mobility of SiC is beneficial to shuttle the charge carriers quickly, which could offer an opportunity to design SiC-based sensors with fast response/recovery time. These unique advantages have led SiC to be regarded as a promising candidate for electro-devices (chemical sensors). As we all know, nanostructured SiC materials with low dimensionality are expected to show excellent properties due to their quantum confinement and morphology effects. Related research has focused on preparing low-dimensionality SiC nanostructures and correlating their morphologies with their size-controlled electrical performances [78]. 

### 4.1. Gas Sensors

Currently, the application of SiC in the field of gas sensors is mainly based on commercial SiC chips, where insulation or oxide layers and precious metals are deposited to form metal insulator semiconductor field-effect type, Schottky diode, P-N junction diode, and metal oxide semiconductor capacitive gas sensors. These sensors are expected to offer satisfactory performance in gas detection in harsh environments such as high temperature and humidity [79]. At present, the detection of gases such as hydrogen [80], nitrogen oxides [81,82], carbon monoxide, and alkanes [79] at high temperatures is being achieved by controlling the insulation oxide layer, noble metal types, and gas sensing models. However, according to the gas response mechanism, the generally long response time of SiC-based gas sensors makes it difficult to meet the critical requirements for practical applications. Thus, the sensitivity and the intrinsic response speed of SiC-based gas sensors still have lots of room for improvement.

One-dimensional SiC nanomaterials, due to their high aspect ratio and specific surface area, are prone to providing more active sites for the target gas. They can quickly cause surface ion transport when the external environment changes, resulting in rapid changes in their electrical properties. Therefore, using one-dimensional SiC nanomaterials to prepare gas sensors is expected to have characteristics such as fast response and high sensitivity, and can be stable when used in extreme environments. To the best of our knowledge, the most widely studied sensors based on SiC material are hydrogen gas sensors.

#### 4.1.1. Hydrogen Sensors

**Figure 9:**
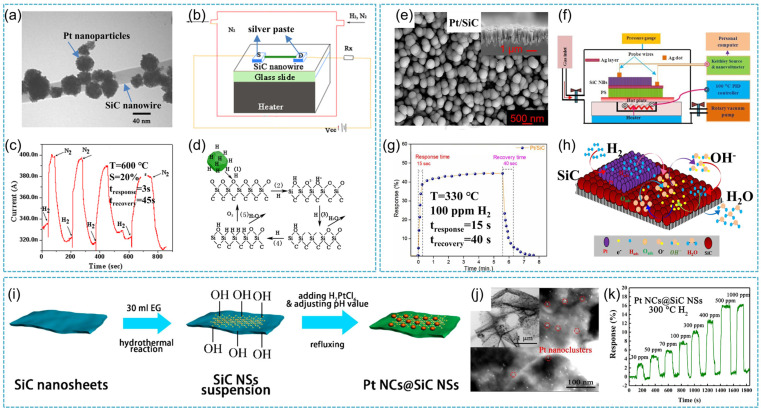
(**a**) TEM images of Pt nanoparticle-decorated SiC nanowire; (**b**) schematic of the Pt-nanoparticle decorated single SiC nanowire sensor and its operation; (**c**) the typical response curve of the SiC nanowire sensor; (**d**) a schematic showing probable hydrogen sensing processes occurring on the Pt nanoparticle decorated SiC nanowire [83]; (**e**) SEM surface morphology of Pt-decorated SiC sensing layer; (**f**) schematic diagram for sensing measurement setup; (**g**) gas response curve vs. time (1–8 min.) for 100 ppm hydrogen gas at operating temperature of 330 °C; (**h**) schematic illustration of hydrogen gas sensing mechanism of Pt decorated SiC nanoballs [84]; (**i**) preparation process of Pt NCs@SiC NSs; (**j**) HAADF-STEM images of Pt NSs@SiC NSs; (**k**) dynamic response curves towards different H_2_ concentrations [85].

As one of the most widely used nanostructures, SiC nanowires can afford an inexpensive and miniaturized alternative for hydrogen sensing. In a typical work, Pt nanoparticle decorated SiC nanowires were fabricated. The obtained SiC nanowires had a diameter of 100 nm and the Pt nanoparticles were distributed uniformly on the SiC nanowire surface (Figure 9a). Silver paste used as the source and the drain electrodes was applied at both ends of the Pt-nanoparticle decorated single SiC nanowire sensor. Schematic of the single SiC nanowire sensor is shown in Figure 9b, which exhibited high sensitivity (S = 20%) and fast response/recovery time (3 s/45 s) toward H_2_ at 600 °C (Figure 9c). It was found that hydrogen atoms can react with the surface oxygen of SiC nanowires to form hydroxyl groups (Figure 9d). These groups can react with surface oxygen to form water, creating an oxygen vacancy and further contributing to the conductivity of SiC nanowires [83]. In addition, DC/RF magnetron sputtering was applied to synthesize Pt-decorated SiC nanoballs (Figure 9e) and their gas-sensing properties toward H_2_ at a high operating temperature range (30–480 °C) studied (Figure 9f). The sensor exhibited a high sensing response (S = 44.48%) with a very fast response time (15 s) toward 100 ppm at 330 °C (Figure 9g). It was found that the response time decreased with rising concentration and the recovery time increased with increased H_2_ concentration, mainly due to the diffusion-limited kinetics at low H_2_ concentration. The high active surface area of SiC nanoballs affords a large number of catalytically enriched surface reaction sites for H_2_ adsorption (Figure 9h), which could enhance the hydrogen detection rate. It affords a new method to design and construct a promising gas sensor with excellent properties for low detection of hydrogen in harsh environments [84]. Pt nanoclusters@SiC nanosheets were produced via a simple one-step wet chemical reduction reaction (Figure 9i) by Sun et al. [85]. The result showed that Pt clusters with a diameter of 2–3 nm were homogeneously distributed on the surface of SiC nanosheets (Figure 9j). The Pt NCs@SiC NSs showed a good response (15.7%) towards 500 ppm H_2_ at 300 °C and this novel device exhibited good stability over a month and a good linear relationship between response and H_2_ concentration (Figure 9k). It affords a simple large-scale preparation method to synthesize hydrogen sensors applied to high-temperature harsh environments. The Schottky junction between Pt and SiC plays an important role in improving gas sensing properties. In addition to noble metal decorated SiC composites, other SiC coupled semiconductors as gas sensing material were also synthesized. Rýger et al. [86] demonstrated that the GaN/SiC heterostructure exhibited enhanced sensitivity towards H_2_. The sensor device showed a low detection limit (20 ppm) and a short response time (12 s). It offers a new strategy for fast online gas analysis systems. 

#### 4.1.2. Other Gases Sensors

SiC-based sensors can detect not only hydrogen but other gases as well. For example, SiCNT has high reactivity due to its sp^3^ hybridization and polarization characteristics of silicon atoms, even higher than CNT [87]. Theoretical investigations show that the adsorption of gas molecules by SiCNTs is mainly chemical, rather than the physical adsorption mechanism of CNTs, which results in the target gas having a greater impact on the surface and bulk electrical properties of the SiCNTs. Therefore, the SiCNTs have a faster response speed to gases. In this regard, researchers predicted through theoretical calculations that SiCNT has good gas sensing performance for gases such as CO [88], HCN [88], CO_2_ [89], O_2_ [87], NO_2_ [90], HCHO [91], SO_2_ [92], and C_2_H_6_ [93]. Unfortunately, the controlled preparation of SiCNTs is currently difficult, and there have been no research reports on the gas-sensing experimental results of SiCNTs. 

**Figure 10:**
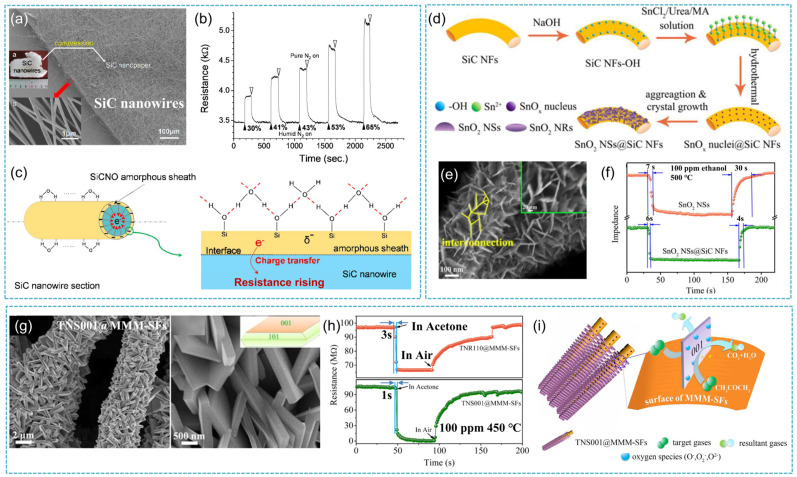
(**a**) SEM images and photographs of SiC nanopaper (inset is the image of nanowires); (**b**) the humidity sensing performance of SiC nanopaper in an atmosphere of different RH; (**c**) the proposed humidity sensing mechanism of SiC nanowire [96]; (**d**) schematic illustration of the synthesis process for SnO_2_ NSs@SiC NFs; (**e**) SEM image of SnO_2_ NSs@SiC NFs; (**f**) comparison of the response time and recovery time between pure SnO_2_ NSs, and SnO_2_ NSs@SiC NFs, ethanol concentration 100 ppm, operating temperature 500 °C [98]; (**g**) SEM images of TNS001@MMM-SFs; (**h**) the response/recovery behaviors toward 100 ppm acetone at 450 °C; (**i**) proposed sensing mechanism for the high response and ultrafast response/recovery rate of the TNS001@MMM-SFs [99].

Researchers have also carried out similar work on the gas sensing performance of other one-dimensional SiC nanomaterials based on the theoretical analysis results of SiCNTs, mainly targeting SiC nanowires and nanofibers [94]. Wang et al. [95] reported the humidity-sensitive performance of SiC nanowires at room temperature for the first time. The study suggests that the physical and chemical adsorption of water molecules on SiC nanowires determines their dielectric constant under different humidity conditions, causing capacitance changes. Li et al. [96] believed that water molecules in the air were ionized when adsorbed on the nanowire surface forming a hydrogen bond. The formation of the hydrogen bonds leads to a slightly negative charge of the sheath since water molecules donate electrons. With the transfer of some electrons from water molecules into SiC nanowires, the resistance of p-type SiC nanowires increases and a humidity-sensitive response occurs (Figure 10c). The SEM morphology of SiC nanowires and the test results under different humidity conditions are shown in Figure 10a and Figure 10b, respectively. ZnO/SiC nanofibers were synthesized via electrospinning of polymer solutions followed by heat treatment. This process is necessary for polymer removal and crystallization of semiconductor materials. The experiment results demonstrated that the ZnO/SiC nanocomposite exhibited a higher concentration of chemisorbed oxygen, a higher activation energy of conductivity, and a higher sensor response towards CO and NH_3_ as compared with ZnO nanofiber [97]. Sultan et al. [32] reported the synthesis of polypyrrole (PPy) and polypyrrole/SiC nanocomposites (PPy/SiC) and PPy/SiC/dodecylbenzenesulfonic acid (DBSA) by an in situ chemical polymerization method and their application was used as a sensor for the detection of highly toxic chlorine gas. PPy/SiC/DBSA nanocomposite was found to possess higher DC (direct current) electrical conductivity as compared to that of Ppy and Ppy/SiC. The sensing response was determined based on the change in DC electrical conductivity. The responses of PPy and both the nanocomposites were found to be highly sensitive and reversible to chlorine gas. This seems to be promising as an effective approach towards the sensing technology for the detection of chlorine gas. 

Our group Wang et al. [98] reported an effective approach to fabricating SnO_2_@SiC hierarchical architectures by a hydrothermal process by growing SnO_2_ nanosheets on a SiCNF surface (Figure 10d,e). The unique SnO_2_@SiC hierarchical architectures could strengthen the gas sensing performance of NFs at 500 °C, with a response and recovery time of 4 s and 6 s for 100 ppm ethanol, respectively (Figure 10f). The hierarchical architectures have a significant impact on the rapid response and recovery of SiCNFs. Subsequently, the facets-exposed TiO_2_ nanosheets and facets-exposed TiO_2_ nanorods were successfully grown on the surface of macro-meso-microporous SiC fiber (MMM-SF) (Figure 10g). The obtained TiO_2_/SiC composites possessed a core-shell hierarchical structure, which exhibited a high response time of only 1 s at 450 °C towards 100 ppm acetone (Figure 10h). The excellent gas sensing properties are due to the exposure of high-energy {001} crystal of TiO_2_ and the synergetic effect of TiO_2_/SiC heterojunctions as well as the core-shell hierarchical architectures. As shown in Figure 10i, acetone molecules could be absorbed and desorbed on the {001} facets-exposed TiO_2_ nanosheets (TNS001) from a discretional direction almost without any hindrance due to the vertical growth of TNS001 on the MMM-SF [99]. Recently, Wu et al. [100] prepared multi-level structured SiCNFs composed of SiC nanorods wrapped together through electrospinning technology combined with a high-temperature pyrolysis process and then loaded Pt nanoparticles with a diameter of 2–3 nm on the fiber surface through the ethylene glycol reduction method to study their high-temperature ammonia performance. The multi-level structure SiCNFs loaded with Pt nanoparticles exhibited a sensitivity of 9.1% to 500 ppm ammonia gas at 500 °C, with a response and recovery time of only 2 s and 5 s, respectively, demonstrating good high-temperature ammonia sensitivity. Table 3 shows the typical gas sensing performances of SiC-based sensors. 

### 4.2. Pressure Sensor

High-temperature pressure sensors have applications in many domains, such as advanced industrial, automotive, and aerospace [108], utilizing this parameter to keep the equipment healthy and running. Particularly, they play an essential role in monitoring the fuel efficiency in the combustor hot zone to reduce emissions and improve reliability [109]. To achieve this aim, pressure-sensitive materials and temperature-resistance are required. Therefore, SiC is particularly viewed by researchers because of its outstanding properties. In terms of mechanical properties, SiC has a higher stiffness and fracture strength as well as better resist wear, oxidation, and corrosion than commonly used silicon [110]. There are two main types of pressure sensors: one type utilizes capacitive effects in sensing pressure and the other type uses piezoresistive effects. Researchers have conducted a lot of work in both areas.

**Figure 11:**
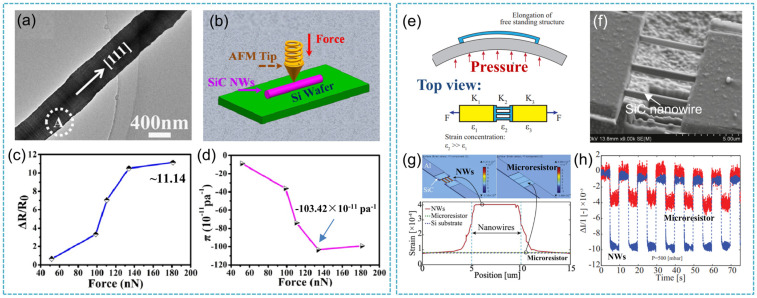
(**a**) TEM images of as-synthesized SiC nanowires; (**b**) schematic diagram for the measurement of the piezoresistance effect in SiC nanowire; (**c**) the ca. ΔR/R0 ratios as a function of the applied forces; (**d**) relationship between the, ca., piezoresistance coefficients and the applied lading forces [113]; (**e**) schematic sketch of strain induced into the free standing nanowire and micro bridge under pressure; (**f**) SEM image of the nanowire array from the top view, showing the nanowires are connected to two serial micro resistors; (**g**) simulation of strain induced into the nanowires and microscaled frames; (**h**) a 3-fold increase in the sensitivity of nanowire sensors (blue) in comparison to SiC microresistors (red) [115].

Capacitive-type pressure sensors are attractive for high-temperature applications because the device performance is tolerant of contact resistance variations and wireless sensing schemes can be readily realized to eliminate any potential performance degradation due to wiring parasitic capacitances. Furthermore, capacitive devices can achieve high sensitivity, low turn-on temperature drift, and minimum dependence on side stress and other environmental variations [108]. Marsi et al. [111] prepared a 3C-SiC diaphragm capacitive pressure sensor and tested the capacitance under different pressures and temperatures. Compared with Si-based capacitive pressure sensors, 3C-SiC-based capacitive pressure sensors exhibited good thermal stability and high sensitivity. At conditions of 1000 °C and 100 MPa, the capacitance was 70 pF. For piezoresistive type pressure sensors, piezoelectric materials exhibit unique electromechanical coupling and have recently received growing interest in the miniaturization of electromechanical devices down to micro/nano scales. These novel materials are particularly attractive for applications in energy harvesting, sensing, actuating, etc., due to their unique advantages such as small size, high sensitivity, high stability, low cost, and a simple readout circuit. For example, the piezoresistance behaviors of single-crystalline n-type 3C-SiC nanowires with an N doping level of 8.28 at% were investigated [112]. The sensitivity was 7.7 × 10^−11^ Pa^−1^ under a load of 135.25 nN. Subsequently, P-type 3C-SiC nanowires with B dopants were synthesized by catalyst-assisted pyrolysis of polysilazane. As shown in Figure 11a,b, the transverse piezoresistance measurement of an individual 3C-SiC nanowire was performed under AFM at RT. Compared to those of conventional SiC materials (bulk 3C-SiC often <0.2, p-type 3C-SiC thin film often <0.03), the ca. Δ*R*/*R*_0_ of p-type 3C-SiC nanowires was much higher up to 11.14 (Figure 11c), suggesting that the resistance changes of SiC nanowires with B dopants were much more sensitive than those of conventional SiC materials. Interestingly, the piezoresistance coefficient π [109] of the wire changed from −8.83 to −103.42 × 10^−11^ Pa^−1^ as the loaded forces varied from 51.7 to 181.0 nN (Figure 11d); the corresponding GF was up to −620.5, suggesting their promising applications in pressure sensors with high sensitivities [113]. Subsequently, in a similar way, Prakash et al. presented the fabrication of SiC nanowires with co-doped N and P elements, which were fabricated via the pyrolysis of a polymeric material [114]. The measured transverse piezoresistance coefficient of the established SiC nanowires increased from 5.07 to −146.30 × 10^−11^ Pa^−1^ as the loading forces varied from 24.95 to 130.51 nN. The corresponding GF was calculated up to ca. −877.79, which was higher than the values for all SiC nanostructures that had ever been reported.

Phan et al. investigated the strain concentration effect of nanowires by both theoretical and experimental routes (Figure 11e,f) [115]. Based on nanowires locally fabricated on free-standing structures with a high strain concentration, the strain induced into nano-scaled sensing elements is amplified while the bulk materials are still at a small strain regime, thereby enhancing the sensitivity of the sensors. The strain induced into the as-fabricated nanowires was derived to be approximately five times larger than that of the microresistors (Figure 11g). The response of the nanowire pressure sensors was approximately three times larger than that of the pressure sensors using micro-sized SiC (Figure 11h). In addition, for the SiC fibers in unidirectional glass/epoxy composites, the resistance change of the SiC fibers was measured according to the applied mechanical strain. In this study, the strain sensing characteristics of semi-conductive SiC fibers were investigated and the electrical sensing properties of the SiC fibers were evaluated. As a result, the piezoresistivity of the SiC fibers showed outstanding strain sensitivity with an average GF of 8.25 and excellent linearity up to the strain range of 1.36% [116]. From this, it can be seen that compared with the research on bulk SiC, one-dimensional SiC nanostructures have shown potential application prospects in the field of pressure sensors.

In situ electrical measurement experiments in individual SiC nanowires were carried out for tensile strain using a transmission electron microscope. Fracture strain approaching 10% was achieved for a diamond-structure SiC nanowire with a <111> direction. The calculated piezoresistance coefficient of this SiC nanowire was −1.15 × 10^−11^ Pa^−1^, which is similar to the coefficient of the bulk material [117,118,119]. Pulliam et al. [120] developed a micromachined SiC fiber optic pressure sensor for use in the extreme temperatures and pressures of propulsive environments. Meanwhile, optical signal processing using sapphire waveguides was developed for this application. The combination of the sapphire waveguide and a SiC membrane chip provides a fiber optic pressure sensor capable of operating above 1100 °C. Ultrasonic vibration mill-grinding was applied to fabricate the SiC diaphragm with a thickness of 43 μm and a surface roughness of 19 nm. The sensor head was formed using a nickel diffusion bonding technique. The pressure sensor shows good linearity in the range of 0.1–0.9 MPa, with a resolution of 0.27% F.S. at room temperature [121]. The electron emitter of the individual SiC nanowire was placed and soldered using the electron beam-induced carbon deposition technique. The results demonstrate that the piezoresistive effect caused by the electrostatic force had a significant impact on the electronic transport properties of the nanowire, and excellent electron emission characteristics can be achieved in the pulse voltage driving mode, including lower turn-on voltage and higher maximum current [122]. The propagation of the Lamb modes along a-SiC/c-ZnO thin supported composite structures by different ZnO and a-SiC layer thicknesses and electrical boundary conditions was simulated by Caliendo. A pressure sensitivity of 9 ppm kPa^−1^, in the 4–10 kPa range, was predicted for the a-SiC/ZnO ZGV (Zero Group Velocity) based pressure sensor [123]. A SiC and aluminum nitride-based DTMCPS (Double Touch Mode Capacitive Pressure Sensor) with a substrate notch was explored. Condensed yet exhaustive step-by-step mathematics of key performance parameters were detailed for the sensor under study. This was carried out to provide a detailed understanding of the underlying physical and mathematical principles [124], and aimed to provide a fast analysis model for prototyping the sensor. Nakamura et al. [125] simulated strain gauge factors in several n-type alpha and beta SiC nanosheet models based on first-principles calculations. Their original procedure of simulating piezoresistive properties was applied to the two-dimensional system with a multivalley conduction-band structure. The calculated gauge factors of the 2H-SiC (0001) nanosheet model for the [1,2,3,4,5,6,7,8,9,10,11,12,13,14,15,16,17,18,19,20,21,22,23,24,25,26,27,28,29,30,31,32,33,34,35,36,37,38,39,40,41,42,43,44,45,46,47,48,49,50,51,52,53,54,55,56,57,58,59,60,61,62,63,64,65,66,67,68,69,70,71,72,73,74,75,76,77,78,79,80,81,82,83,84,85,86,87,88,89,90,91,92,93,94,95,96,97,98,99] tensile strain were very small at room temperature, but the longitudinal gauge factor showed a significant negative value at high temperatures. Moreover, microstructures on sub-100 nm SiC membranes with a large aspect ratio up to 1:3200 play an important role in response [126]. Unlike conventional processes, this approach started with Si wet etching to form suspended SiC membranes, followed by micro-machined processes to pattern free-standing microstructures such as cantilevers and micro bridges. The authors demonstrated a SiC pressure sensor by applying lithography and plasma etching on released ultrathin SiC films. The sensors exhibited excellent linear response to the applied pressure, as well as good repeatability. Table 4 summarizes recently developed, typical SiC-based pressure sensors from the perspectives of materials, temperature, pressure, and sensitivity (or gauge factor, piezoresistance coefficient).

### 4.3. Bio-Sensors

In addition to excellent electronic and mechanical properties, SiC also has the characteristics of biocompatibility, versatility, chemical stability, and transparency to visible light, making it suitable for bio-sensor applications. For example, SiC nanomaterials can be applied in the detection of DNA molecules, organophosphate (OP) molecules, and nitrite, etc. For DNA detection, Fradetal et al. [132] functionalized two types of SiC nanopillar arrays; one was top-down SiC nanopillars (pitch: 5 μm) and the other one was a dense array (pitch: 200 nm) of core-shell nanopillars. Depending on both the pillar morphology and the pitch, different results in terms of DNA surface coverages were obtained. Particularly, the DNA molecule coverage was not similar from one nanopillar array to another, which depended on the case of wide-pitch array. It was concluded that to achieve a DNA sensor based on a nanowire-field effect transistor, the functionalization must be conducted on a single SiC nanowire or nanopillar that constitutes the channel of the field effect transistor and be further experimentally verified. Subsequently, SiC nanowire field effect transistors were synthesized and functionalized with DNA molecule probes via covalent coupling using an amino-terminated organ silane. The experimental results demonstrated the current of the sensor was lowered by 22% after probe DNA grafting and by 7% after DNA hybridization [133].

**Figure 12:**
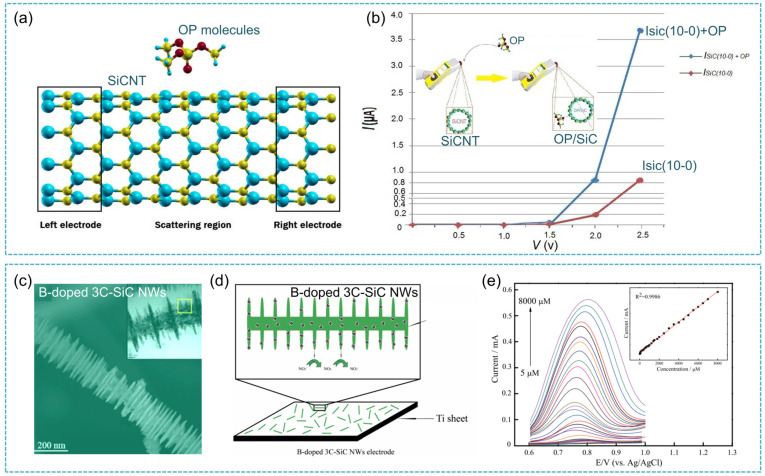
(**a**) A schematic illustration of SiC nanotube-based sensor for detecting OP molecule; (**b**) the calculated I-V curve before and after the adsorption of OP molecule on SiC nanotube [134]; (**c**) SEM image of the B-doped cubic SiC nanowires; (**d**) the schematic illustration of electrochemical detection of nitrite based on B-doped cubic SiC nanowires electrode; (**e**) DPV recordings of nitrite at B-doped cubic SiC nanowires electrode in PBS (0.1 moL·L^−1^) with different nitrite concentrations [135].

For organophosphate molecules, Hassanzadeh et al. [134] found that the OP molecules could be adsorbed at silicon sites of SiCNTs (Figure 12a). It can be concluded that a strong bond formed between the OP and SiC nanotube. The ΔE_gap_ showed many changes (12%) in electronic properties, which could induce alteration in the SiCNT electrical conductivity (Figure 12b). In addition, electrochemical nitrite sensors based on cubic SiC nanowires with smooth surfaces and boron-doped cubic SiC nanowires with fin-like structures were reported for the first time (Figure 12c) [135]. As for the electrochemical behavior of both SiC nanowire electrodes, the cyclic voltametric results showed that both SiC electrodes exhibited a wide potential window and excellent electrocatalytic activity toward nitrite oxidation (Figure 12d). There existed a good linear relationship between the oxidation peak current and the concentration in the range limitation of 50–15,000 umol·L^−1^ and 5–8000 umol·L^−1^ (Figure 12e) with the detection limitation of 5 and 0.5 umol·L^−1^, respectively. In addition, a continuous glucose sensor employing radio frequency signals using the biocompatible material SiC was successfully fabricated [136]. To test the sensor as a function of glucose level, changes in sensor performance to varying glucose levels were measured and a shift in resonant frequency to lower values was observed with increasing glucose level. The functionalization of SiC for biosensing applications was demonstrated by Williams [137]. 4H-SiC was functionalized with 3-aminopropyltriethoxysilane (APTES) and subsequently biotinylated for the selective immobilization of streptavidin. The experimental results demonstrated that the APTES functionalized and biotinylated SiC surface had the potential to be employed as a biosensing platform for the selective detection of streptavidin molecules.

### 4.4. Other Sensors

**Figure 13:**
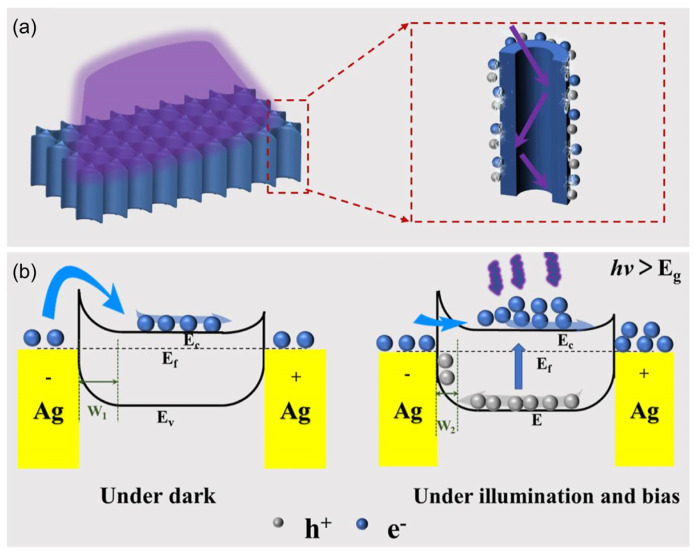
(**a**) Schematic illustration of the enhanced absorption capacity of the 4H-SiC nanohole arrays. (**b**) Schematic diagram of the energy band of the Ag-4H-SiC-Ag MSM photodetector in dark and light illumination conditions [145].

Placidi [138] combined the stable performance of 3C-SiC under high-frequency conditions to prepare an oscillator in a terahertz environment, which improved its performance by 50% compared with the commonly used Si-based sensors. Dakshinamurthy et al. [139] found that the refractive index of 6H-SiC for 632.8 nm He Ne laser varies with temperature, and can be used to prepare wireless temperature sensors in high-temperature and harsh environments. Theoretical calculations by Kumar et al. [140] demonstrated that one-dimensional photonic crystals designed and prepared using 4H SiC and TiO_2_ can be used as temperature sensors. In the same year, Rao et al. [141] prepared Schottky diode temperature sensors using 4H SiC, which can operate stably in the range of 30–300 ºC and have high sensitivity. Moreover, Peng et al. [142] prepared SiC nanowire ultraviolet (UV) sensing sensors using the CVD method. The Sciuto group [143,144] prepared UV photoelectron (PDs) sensors using 4H SiC and 6H SiC, which have a wide spectral detection range. Among them, 6H SiC-based sensors have the potential to achieve visible light detection. Yang et al. [135] prepared highly sensitive SiC nanostrip photoelectric detection sensors, which can work stably at 300 ºC for up to 180 days, indicating their promising application prospects in harsh environments. Recently, a high-performance UV PD with single-crystal integrated self-supporting 4H-SiC nanohole arrays was constructed, prepared via the anode oxidation approach. The PD delivers a high responsivity (824 mA/W), superior to those of most reported ones based on 4H-SiC (Figure 13) [145]. 

## 5. Discussion and Future Trends

In recent years, one-dimensional SiC nanomaterials have attracted great attention due to their unique structure and outstanding properties. Significant progress has been witnessed in one-dimensional SiC nanomaterials in the development and application of sensors. Many methods have been developed to prepare SiC nanomaterials with different morphologies and structures, such as templates, chemical vapor deposition, electrospinning, and carbothermal reduction methods. Although the rapid development of one-dimensional SiC nanomaterials has promoted the exploration of their applications, the research based on one-dimensional SiC nanomaterial sensors is still in its early stages, and many challenges lie ahead.

1. Theoretical calculation results have demonstrated that SiCNT has outstanding gas sensing and other sensing performance, but current experiments are still difficult to achieve an accurate preparation of SiCNT. Preparing and constructing SiCNT-based sensors has been one of the major challenges for future research.
2. The focus of research on one-dimensional SiC nanomaterial sensors has primarily been on preparation and performance, and while these sensors have shown potential, the research on sensing principles has still not been conducted sufficiently indepth. One of the keys to improving the sensing performance and developing high-performance sensors is to explain the sensing principle through the combination of theoretical calculations and advanced analytical characterization methods.
3. Selecting suitable components and designing rational morphology and structure based on application objectives and sensing principles are essential to obtain one-dimensional SiC nanomaterials with the desired sensing performance. Further surface chemical functional treatments, precious metal modifications, and construction of heterostructures can also improve the sensing performance, which can facilitate new processes and approaches for the development of high-performance SiC-based sensors.
4. At present, the research range of one-dimensional SiC nanomaterial sensors is still narrow, and the sensing performance is unitary. How to make full use of the advantages of SiC nanomaterial multifunction and develop one-dimensional SiC nanomaterial multifunction sensors is a new direction of one-dimensional SiC nanomaterial sensor research in the future.
5. SiC is quickly emerging as a versatile material for quantum sensing applications, and integrating SiC color centers into devices based on 1D SiC nanostructures would be able to increase their potential applications in energy-based sensors.

At this point, further intensive research is required to overcome these challenges. With deeper investigations on the sensing principles, fabrication processes, and sensing properties, the one dimensional SiC nanomaterial sensor will be able to satisfy the requirements for practical applications in extreme environments.

## Figures and Tables

**Table 1:** Typical properties of SiC and other semiconductors [6].

	Si	GaAs	3C-SiC	4H-SiC	6H-SiC	Diamond
Lattice (Å)	5.43	5.65	4.36	3.08	3.08	3.567
Lattice (Å)	5.43	5.65	4.36	15.12	10.05	3.567
Bond length (Å)	2.35	2.45	1.89	1.89	1.89	1.54
TEC (10^−6^ K)	2.6	5.73	3.0		4.5	0.8
Density (g cm^−3^)	2.3	5.3	3.2	3.2	3.2	3.5
Thermal conductivity (W cm^−1^ K^−1^)	1.5	0.5	5	5	5	2
Melting point (℃)	1420	1240	2830	2830	2830	4000
Mohs hardness			9	9	9	10
E_g_ (eV)	1.1	1.43	2.3	3.3	3.0	5.4

**Table 2:** Fabrication methods and application of one-dimensional SiC nanomaterials.

Methods	Materials	Nanostructure	Applications	Reference
Template	CNT + SiO	Nanowhisker	--	[7]
	CNT + SiO(SiCl_4_)	Nanorod	Composites	[8]
	CNT+ SiO	Nanotube	Hydrogen storage	[9]
	Silicon nanowires + propylene	Nanoarray	Photoluminescence	[10]
CVD	C + SiO_2_	Nanofiber	--	[11]
	*l*-PCS + carbon powder	Nanowire	Electronics	[12]
Electrospinning	CNF + SiO	Nanowire	Electronics	[13]
	CNF + Si	Nanofiber	Photocatalysis	[14]
	PCS + PVP	Nanofiber	Microwave absorber	[15]
Carbothermal	TEOS + carbon black	Nanowire	Flame-retardant	[16]
	Electronic waste	Nanowire	Photocatalysis	[17]
	Silicone oil	Nanorod	Hydrogen storage	[18]
	Ethanol + SiCl_4_ + lithium	Nanobelt	Photoluminescence	[19]
	SiC + carbon	Nanorod	--	[20]
	TEOS + sugar + aluminum	Nanowire	Photoluminescence	[21]

**Table 3:** Gas-sensing performances of SiC-based sensor nanostructures.

Material	Structure	Target Gas	C (ppm)	Tem.	Response	t_res_/t_rec_	LOD	Ref.
Pt/SiC	nanowire	H_2_	40,000	600	20%	3/45	—	[83]
Pt/SiC	nanoballs	H_2_	100	330	40%	15/40	5	[84]
Pt/WO_3_/SiC	nanolayer	H_2_	10,000	530	—	—	—	[101]
Pd/SiC	nano cauliflower	H_2_	100	300	40%	7/13	2	[102]
Pd/SiC	nanofilm	H_2_	—	—	—	—	—	[103]
Graphene/SiC/Si	nanowalls	H_2_	150	—	25%	—	0.5	[104]
Pt/SiC	nanosheet	H_2_	500	300	15.7%	—	—	[85]
PdPt/SiC	nanofilm	H_2_	100	350	60%	21/35	5	[105]
Pt/WO_3_/SiC	nanofilm	H_2_	2000	350	—	—	—	[106]
Pt/WO_3_/SiC	membrane	H_2_	20,000	—	—	50/50	—	[107]
SnO_2_/SiC	nanofiber	Ethanol	100	500	25	4/6	10	[98]
ZnO/SiC	nanofiber	CO	20	500	110%	—	—	[87]
Pt/SiC	nanofiber	NH_3_	500	500	9.1%	2/5	1	[99]
TiO_2_/SiC	nanofiber	CH_3_COCH_3_	200	450	30	3/12	1	[100]
Ppy/SiC	nanoparticle	Cl_2_	—	—	—	—	—	[32]

**Table 4:** Summary of typical SiC-based pressure sensor and their key performance.

Materials	Temperature/°C	Pressure	Sensitivity	Type	Ref.
**4H-SiC**	23–800	1.38 MPa	5.45	piezoresistive	[109]
**SiC**	300–574	100 psi	7.2 fF/psi	capacitive	[127]
**6H-SiC**	RT-400	60 psi	5.817 mV	piezoresistive	[128]
**3C-SiC/single crystal**	RT-400	1100–1760 torr	7.7 fF/torr	capacitive	[108]
**SiC fiber**	RT	1.36%	8.25	piezoresistive	[115]
**3C-SiC/nanowire**	RT	500 mbar	33	piezoresistive	[114]
**PVDF/SiC nanowire**	SiC nanowires as nucleating agent				[129]
**3C-SiC/single crystal**	RT-500	5 MPa	0.3477 mV MPa^−1^	capacitive	[110]
**3C-SiC nanowire**	RT	153.56 nN	7.7 × 10^−11^ Pa^−1^	piezoresistive	[112]
**a-SiC/c-ZnO**	RT	10 kPa	10.98 ppm kPa^−1^	strain/frequency	[123]
**3C-SiC thin film**	27–1000	100 MPa	70 pF	capacitive	[111]
**PECVD SiC coating film**	RT	1 MPa	17.1 mV V^−1^ MPa^−1^	piezoresistive	[130]
**B/3C-SiC nanowire**	RT	181.0 nN	−103.42 × 10^−11^ Pa^−1^ /−620.5	piezoresistive	[113]
**N/P/SiC nanowire**	RT	130.51 nN	−146.30 × 10^−11^ Pa^−1^	piezoresistive	[116]
**SiC fiber/SiC foam**	RT	pressure drop			[131]
**SiC fiber**	RT	-	5	piezoresistive	[118]
**SiC fiber**	RT		1.6 × 10^−11^ m^2^ N^−1^	piezoresistive	[117]
**SiC nanowire**	RT		−1.15 × 10^−11^ Pa^−1^	piezoresistive	[119]
**SiC fiber/sapphire fiber**	1100 °C				[120]
**SiC fiber-optical**	RT	0.1–0.9 MPa	0.27 F.S.		[121]
